# Design and ex vivo characterization of narrow implants with custom piezo‐activated osteotomy for patients with substantial bone loss

**DOI:** 10.1002/cre2.276

**Published:** 2019-12-31

**Authors:** Holger Wirz, Stefan Teufelhart, Christine McBeth, Robert Gyurko, Serge Dibart, Alexis Sauer‐Budge

**Affiliations:** ^1^ Fraunhofer USA Center for Manufacturing Innovation Brookline Massachusetts; ^2^ Project Group for Resource‐Efficient Mechatronic Processing Machines (RMV) Fraunhofer Institute for Machine Tools and Forming Technologies (IWU) Augsburg Germany; ^3^ Department of Periodontology Boston University Boston Massachusetts; ^4^ Department of Periodontology, Tufts University School of Dental Medicine Boston Massachusetts; ^5^ Department of Biomedical Engineering Boston University Boston Massachusetts; ^6^ Exponent Inc Natick Massachusetts

## Abstract

**Objective:**

Bone augmentation delays implant placement and increases risks due to additional surgeries. Implant systems compatible with reduced alveolar bone volume are required. To design, manufacture, and test a non‐cylindrical dental implant system using piezotomes and custom‐designed matching titanium mini‐implants to address the needs of patients with missing teeth and narrow jawbone.

**Materials and methods:**

Tapered mini‐implants with a rectangular cross‐section (4.6 mm × 2.1 mm) were machined with dimensions that could accommodate narrow alveolar ridges. The performance of the implants were tested in both static and fatigue cycle 30° compression tests. Tapered, rectangular cutting tools that matched the overall trapezoidal morphology of the implant were also designed. These novel tools were engineered to be compatible with commercially available piezoelectric osteotomes. Tools were optimized using finite element analysis and were manufactured accordingly and were used by a periodontal surgery team in a pork rib bone model to monitor utility of the device and ease of use.

**Results:**

The rectangular design of the implant allows for a full occlusal load due to the larger implant flexural rigidity compared to a similar diameter mini‐implant with a standard cylindrical design. During 30° compression fatigue tests, the implant tested at 340 N did not fail after 5M cycles as shown in Kaplan‐Meier survival curves. Finite element analysis allowed for functional optimization of the roughing and finishing tools. In the pork rib model, these tools successfully cut trapezoidal holes that matched the dimensions of the implant.

**Conclusions:**

The implant system here demonstrates the feasibility of a mini‐implant system that has superior flexural rigidity and potentially circumvents the need for patient bone augmentation.

## 1 INTRODUCTION

Tooth loss leads to local resorption of the alveolar bone. This part of the jawbone follows the “use it or lose it” principle. The longer the tooth is missing, the more bone is lost. A recent systematic review of the literature estimates that the average reduction in alveolar bone width is 3.87 mm, and the average reduction of alveolar bone height is 1.67 mm 1 year after tooth extraction (Van der Weijden, Dell'Acqua, & Slot, 2009). The reduction of ridge width is particularly significant, considering that the average ridge width is 9 mm. The current standard of care for missing teeth is implant placement. Current dental implants are cylindrical in shape and are placed using rotary implant burs. Although short implants offer a solution for reduced alveolar bone height (Griffin & Cheung, 2004; Neldam & Pinholt, 2012; Sanchez‐Garces, Costa‐Berenguer, & Gay‐Escoda, 2012; Tutak, Smektala, Schneider, Golebiewska, & Sporniak‐Tutak, 2013), they are not a good match for patients with long‐lost teeth and narrow alveolar ridges. Because of this, the majority of implant patients requires some form of bone augmentation (*Horizontal alveolar ridge augmentation in implant dentistry: A surgical Manua*, 2016; Rammelsberg et al., 2012). Current trends in implantology indicate patients' desire for rapid tooth replacement, as evidenced by the increase in the number of “immediate” implants (placed right after tooth extraction) (Quirynen, Assche, Botticelli, & Berglundh, 2007; Schropp, Kostopoulos, & Wenzel, 2003).

Most dental implants are made of titanium, as this material predictably ankyloses into bone, a process termed *osseointegration*. However, the mechanical properties of titanium places limit on how small the diameter of a cylindrical implant can be while withstanding biting forces. Therefore, cylindrical implants with smaller than 3‐mm diameter, the so‐called “mini‐implants,” cannot be used for tooth restoration but only for other ancillary purposes such as temporary anchoring of orthodontic appliances (Consolaro & Romano, 2014; Flanagan & Mascolo, 2011).

When implants are not placed immediately, bone augmentation with cadaver bone is the most common solution to enlarge the jawbone at the desired implant site. More than 50% of patients requires bone augmentation before or during dental implant placement (Rammelsberg et al., 2012). While generally successful, bone augmentation leads to additional costs and increased treatment time, with an average of 6 months before the bone graft heals and integrates. Additionally, patients are often reluctant toward the use of cadaver bone, and the extra procedure exposes patient to additional surgical risks.

An available alternative to bone grafting in patients with narrow alveolar bone is to forego implant restoration altogether and restore missing teeth with a bridge or removable denture. These solutions, however, have several shortcomings, such as the need for placing crowns on neighboring teeth for a bridge restoration or the discomfort, inferior esthetics, and negative patient perception associated with removable dentures. While bridges and removable dentures are acceptable in some situations, they are no longer the standard of care for missing teeth.

A useful technology in implant surgeries is based on piezo‐driven tools. A piezotome is a miniature bone saw vibrating at ultrasonic frequencies and submillimeter amplitudes. It is currently used in bone surgery for small and precise cuts and in areas where sparing soft tissues is critical. Piezoelectric cutting tools operating at frequencies between 25 and 30 kHz are unable to cut soft tissue but are able to make precise cuts into mineralized bone (Otake, Nakamura, Henmi, Takahashi, & Sasano, 2018). This has been shown clinically to reduce obscuring of the cut by blood and is projected to improve future osseointegration of implants (Landes et al., 2008). An additional benefit of the piezotome is that it increases bone density, thus providing additional support for the implant. The increase in bone density after piezotomy has been observed on clinical computed tomography scans of human subjects undergoing piezotomy for orthodontic purposes (Gyurko & Kim, 2013).

To address the shortcomings of currently available implants, we have developed a PiezoImplant system based on the notion that the implant should match the shape of the available bone, thus eliminating the need for bone augmentation. The essential feature of the piezotome is that it is capable of creating various shapes of nonround bone cuts, as opposed to currently available implant drills that are all rotating instruments. By circumventing bone augmentation, we envision that our PiezoImplant would shorten treatment time by 6 months; present a minimally invasive option to patients; provide significant cost savings; require less specialized skill to implant; and offer increased implant stability due to the observed increased bone density around piezotome cuts in alveolar bone of orthodontic patients. Here, we present the design of the implant and piezo cutting tools, fatigue testing of the implant, and performance of the cutting tools.

## 2 METHODS AND MATERIALS

### 2.1 Implant materials and manufacturing

The implants and abutments were fabricated from Grade 5 Titanium, which is an alloy of titanium with 6% aluminum and 4% vanadium, also known as Ti6Al4V (Alliant Materials, Inc.). This material has a yield strength of 880 MPa and a surface hardness of 36 Rockwell C in the annealed state.

### 2.2 Finite element analysis

Both the implant and the tools were simulated using finite element modeling. The implant was modeled using the known geometry and material properties. For the model of the complex bone structure, a combination of two materials was used. For both materials—the elastic cancellous bone (core of the bone) and the comparably stiff cortical bone (outer shell of the bone)—homogenized materials were defined, which represent the physical properties of the complex structure of the respective bone portion. The corresponding parameters were used:

- Cancellous bone: Cancellous bone consists of a complex lattice structure, which is adapted to the occurring stresses inside the material. For this model, the complex structure was homogenized. The used isotropic material has a Young's modulus of 0.385 GPa and a Poisson ratio of 0.12.
- Cortical bone: Cortical bone is a dense shell around the cancellous bone, which has the task to transfer external loads into the bone. Compared with cancellous bone, a stiffer isotropic material was defined. The Young's modulus is 12.5 GPa and Poisson ratio is 0.3.
- Crown: The crown, which transfers the external forces (e.g., biting forces) into the implant, was modeled by an ideally stiff element (RBE2 element in NX NASTRAN). The abutment was disregarded.
- Implant: For the implant, the known material properties were used.

**Figure 1:**
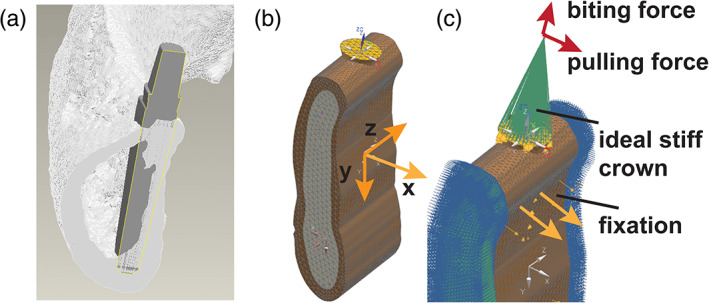
Loads and constraints on the finite element analysis. (a) Model of the implant with well‐known geometry and material parameters embedded in a core of elastic cancellous bone surrounded by a shell of stiff cortical bone. (b) Finite element analysis simulation with axes depicted. (c) Detail of simulation in (b) with biting force, pulling force, ideal stiff crown, and fixation highlighted

The forces were as follows: A biting force of 150 N distributed on four teeth, a pulling force of 50 N distributed on four teeth, and a combination of biting and pulling forces. The bone was fixed by interpolating elements (RBE3 element in NX NASTRAN), which generally constrain the bone section but allow a deformation of the constrained cutting plane. Furthermore, a symmetric constraint was applied on the cutting faces. The simulation model is shown in Figure 1. The FEM problems for the standard 3‐mm round implant and our new implant geometries were solved and compared.

The goose neck design of the tool was optimized using finite element analysis. The initial geometry (straight configuration) and material parameters were modeled, and the dynamic behavior of the tool determined includes modes, frequency response functions, and transmissibility. As the Satelec handpiece operated at 30 kHz, it was desired to tune the neck shape to design a tool with a natural frequency of 30 kHz for the relevant mode. We varied the length of the beam sections, the bending angles, and the bending radiuses to generate a sawing motion at 30 kHz.

### 2.3 Implant manufacturing

The implants were fabricated using conventional milling and turning techniques. The first process step was to rough machine the abutment side of the implant on a CNC lathe (Hardinge super‐precision lathe T51SP). The interface of the abutment and implant was designed with a self‐locking taper angle to accomplish retention and required a smooth surface. Therefore, the second process step was to finish turn these two engineering surfaces (Hardinge super‐precision lathe T51SP), optimizing for best surface finish (diametrical tolerance 0.002 mm). The implant‐bone interface was also tapered for primary stability, but the surface was not machined smooth. In the final machining process, the lower part of the implant was milled on a Bostomatic 1,000 5‐axis milling machine. The ridges created by the milling process on the lower part of the implant were designed to assist in osseointegration (Alla et al., 2011). The ridges were created by using a 1.587‐mm ball end mill (d_1_) with a 0.254‐mm step‐over distance (a_e_) between tool paths. The theoretical peak height created this way is 0.010 mm (R_th_). After masking the implant/abutment interface, the implant surface was treated with an MCD apatitic abrasive and postblasting passivation process (known in the industry as RBM, SBM, and RBT), which is generally recommended for implant surfaces to aid the osseointegration process (HiMed) (Barfeie, Wilson, & Rees, 2015a; Smeets et al., 2016).

### 2.4 Implant fatigue testing

Static and fatigue tests were performed by Engineering Materials Laboratory, Inc. (Sante Fe Springs, CA) on 15 implant/abutment assemblies. All testing was conducted at room temperature (75 ± 5°F) in air, holding the implant/abutment system at a 30° angle relative to the applied load. Instruments used for fatigue testing included the 20,000‐lb capacity Instron Universal Testing Machine (Model TTD ECN 1058) using 200‐lb ECN 1037 full‐scale range, Krouse axial fatigue machine (Model ECN 1087), and a strain gage load cell (S/N C7254) with an Ellis Associates Model BA‐13 bridge/amplifier for dynamic load readout.

### 2.5 Implant/abutment assemblies

The tapered test abutments were preinstalled on the implants by a Boston University personnel. The implants were then potted into acrylic sleeves (modulus of elasticity of 3.2 GPa). The pot line was 3.0 mm below the grit blast termination line on the implant representing a 3‐mm bone recession. A test fixture was used to hold the implant/abutment system 30° off‐axis, per ISO 14801 with the long side of the rectangle (implant surface) in tension/compression. The spherical end cap was incorporated into the abutment such that the center of the contact point was 11 mm above the holding line of the implant measured along the axis of the implant as specified in ISO 14801 Second Edition 2007‐11‐15 Test Method. The test load was applied through a rod that was pin loaded using a center drill point. This allows unconstrained motion in the transverse direction and does not reduce the magnitude of the applied bending moment. The same test fixture was utilized for both static load and fatigue tests.

### 2.6 Static and fatigue 30° compression testing

An Instron Universal Testing Machine, Model TTD with a 200‐lb capacity load range, and 1.27 mm/min (0.05 in/min) crosshead speed was used for the static tests of three implant/abutment assemblies. Once the maximum static load was determined, fatigue tests were run at 80% of the failure load (in triplicate) according to standard industrial protocols (ISO 14801). The test speed was set to 15 cycles per second (Hz), and tests were terminated at 5 M cycles if no failure was observed. Three additional loads were tested in triplicate. Minimum fatigue load was set to 10% of maximum fatigue load.

### 2.7 Tool design and manufacture

The cutting tools were designed to work with the Satelec Piezotome 1. The tools were designed to minimize the number of required tools and thus reduce surgical time. The osteotomy process was envisioned to be conducted with three tools. First, a primary hole is drilled using a standard dental bur (2.0 mm), then a roughing tool is used to shape the initial hole, and a finishing tool to widen and finalize the shape of the hole to match that of the implant. The ultrasonic cutting tools were manufactured in a multistep process. Tool blanks were fabricated using a conventional turning process with a Hardinge SUPER‐PRECISION T high‐precision lathe. The shape of the blanks had final dimensions for the threaded handle interface, and the cross section of what in the final tool would become the goose neck. In the still straight state, the final geometry of the cutting head was produced using a wire electrical discharge machining process. A custom fixture was designed to hold the tool in the various compound angles to produce the complex tooth geometries required for the design. Lastly, the tools were bent to closely resemble the goose neck shape of the simulation results. Like the electrical discharge machining cutting process, a custom fixture was created to facilitate the various holdings and bend angles required to produce the final tool geometry.

### 2.8 Ex vivo testing in rib bone

Primary stability was measured by installing implants into pork rib bone purchased at the supermarket. The rib bones were secured with a vise. Osteotomies were performed by periodontal surgeons (SD and RG). The implants were placed in the osteotomies with a rubber mallet, and their primary stability was gaged by touch.

## 3 RESULTS/DISCUSSION

### 3.1 Design of implant

**Figure 2:**
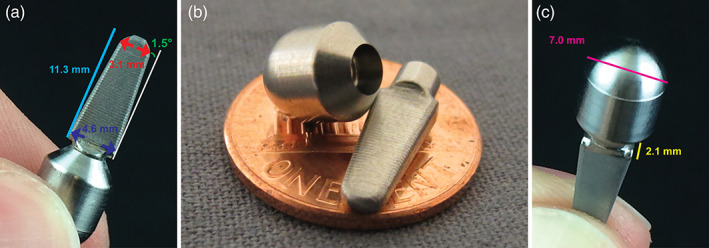
Photograph of the custom designed and in‐house machined PiezoImplant. (a) The length of the implant from tip to shoulder is 11.3 mm with tip width of 3.1 mm and shoulder width of 4.6 mm. (b) Photograph of implant and cap. (c) Cap diameter is 7.0 mm with an implant thickness of 2.1 mm

The implant shown in Figure 2 was designed to be implanted into a narrow ridge of a recessed jaw bone. The cross section of the implanted body is rectangular in shape, resembling the geometry of the bone ridge more closely than a cylindrical implant. A rectangular implant has the further advantage of a higher area moment of inertia than a cylindrical implant of the same diameter (*I*_*y rec*_ *=* 17.0 mm^4^ vs. *I*_*y cir*_ = 0.9 mm^4^ for the implant in Figure 2). This results in a greater flexural rigidity within the narrow ridge of bone for rectangular implants. This rigidity, in conjunction with the increased surface area of the rectangular implant, is expected to improve downstream osseointegration. Another critical feature is the tapered shape of the implant. We used angles similar to Morse tapers. The intent of the Morse taper is for the implant to seat securely in the bone, even before osseointegration occurs. Additionally, ridges that are artifacts of the machining process are expected to improve primary stability immediately after implantation and to increase the surface available for osseointegration for final stability (Figure 2a) (Barfeie, Wilson, & Rees, 2015b).

The upper part of the implant is also shaped like a Morse taper (~1.5°), connecting the implant to the abutment. Although most cylindrical implants use an internal hexagonal connection with a screw to secure the abutment, the small dimensions of our implant did not allow for this type of fixation. An external locking Morse taper design, similar to that used in Bicon dental implants, was used instead. The abutment has a cylindrical shape, topped by a semisphere. This shape facilitates the fatigue testing protocol, allowing the introduction of off‐angle forces with the same moment arm with respect to the implant.

**Figure 3:**
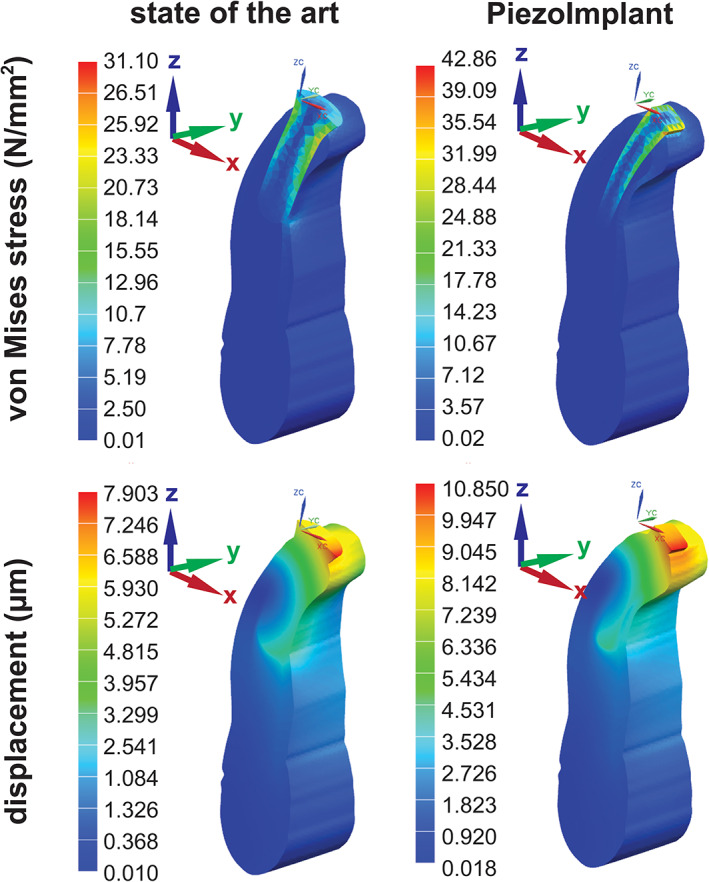
Finite element analysis comparison of PiezoImplant with state‐of‐the‐art implant. Top: von Mises stresses for each simulation (N/mm^2^). Bottom: Absolute displacement for each simulation (μm). Note scale values

With this design, we compared the maximum von Mises stresses and displacement before failure by finite element analysis (Figure 3). For these models, we compared a state‐of‐the‐art implant with the PiezoImplant. Both the maximum von Mises stresses (42.65 vs. 31.12 MPa) and the displacement (10.805 vs. 7.903 μm) were found to be 37% higher for the new design versus the state‐of‐the‐art round implant.

### 3.2 Implant fatigue testing

With the manufactured PiezoImplant, we then set out to determine the forces that caused the PiezoImplant to fail. Also of note is to identify where in the assembly failure occurred. The text fixture included the PiezoImplant with cap abutment embedded in acrylic. Forces were exerted on the assembly at a 30° angle to recapitulate biting force angles. Static compression tests protocols involved increasing the load until implant failure and recording that maximum load. A second round of tests monitored compression fatigue by exposing the assembly to set forces over numerous cycles and identifying the cycle number of failure for each force tested according to industry standards. Results from the static failure load testing showed that the mean maximum compressive load at failure is ~600 N. The maximum loads prior to failure are listed in Table 1.

**Table 1:** Static compression test results

Sample ID	Max load (lbf)	Max load (N)	Failure mode^a^
829	136.7	608	1
1,953	131.9	587	1
2,259	131.8	586	1
Average	133.5 ± 2.8	593.7 ± 12.4	

a Yielding of implant at pot line, Figure 4.

For compression fatigue tests, PiezoImplants were embedded in acrylic and tested at 30° as before. Forces were cycled between maximum and minimum loads with the minimum load set at 10% of the maximum load as detailed in Table S1 **(Boggan, Strong, Misch, & Bidez,** 1999 **)**. At 340 N, 5 M cycles of loading were completed without implant failure. Increasing the maximum load to 400 N and beyond generated implant cracking below the pot line and is depicted a Kaplan–Meier survival plot (Figure 4a). The typical failure mode at these higher forces is also shown (Figure 4b).

**Figure 4:**
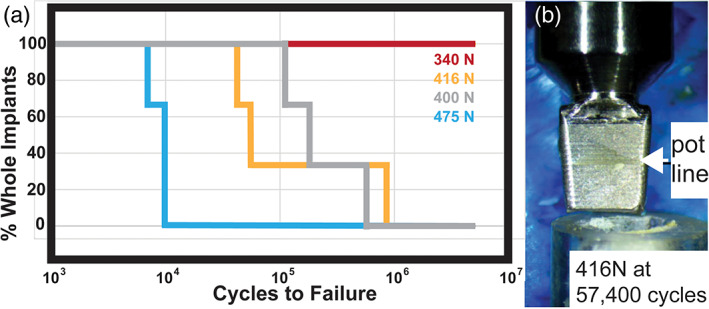
30° compression fatigue tests. (a) Graph showing the cycles to failure for the 30° compression fatigue tests of 12 implant/abutment assemblies. Note that the three assemblies tested at 340 N did not fail after 5 M cycles. *n* = 3 for each load indicated. (b) Photograph showing the fatigue test failure mode with the implant crack below the pot line

### 3.3 Design of tool, FEA results

**Figure 5:**
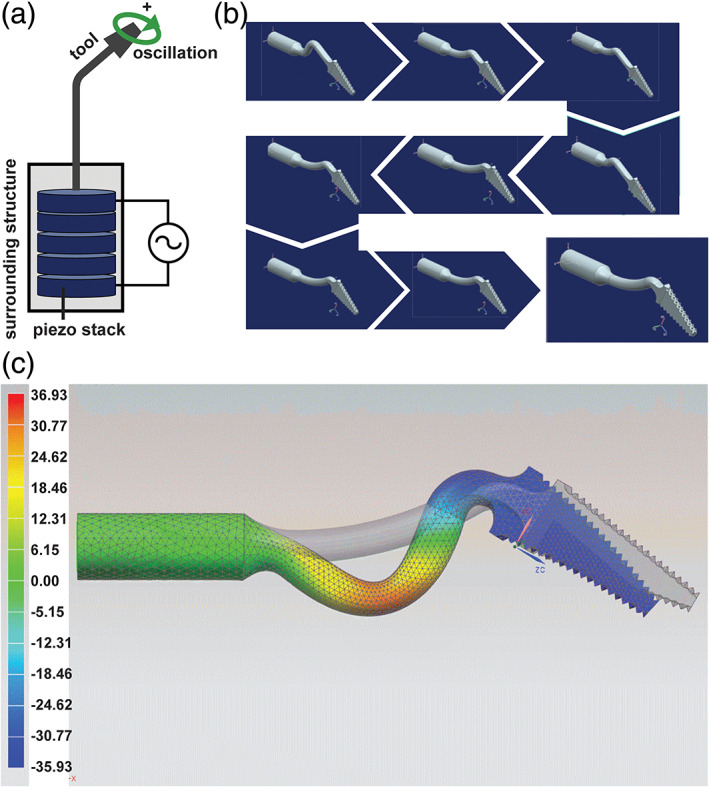
Finite element analysis for optimizing tool shape to reduce oscillations at the tip that would result in erratic cutting. (a) Diagram of piezo stack and tool, highlighting the direction of potential tip oscillations. (b) Progression of tool optimization simulations. Parameters varied included length of the beam sections, bending angles, and bending radii. (c). The final design of the roughing tool (gray) with maximum deformation and deflection for the given excitation (3‐μm axial displacement with a frequency of 30 kHz)

**Figure 6:**
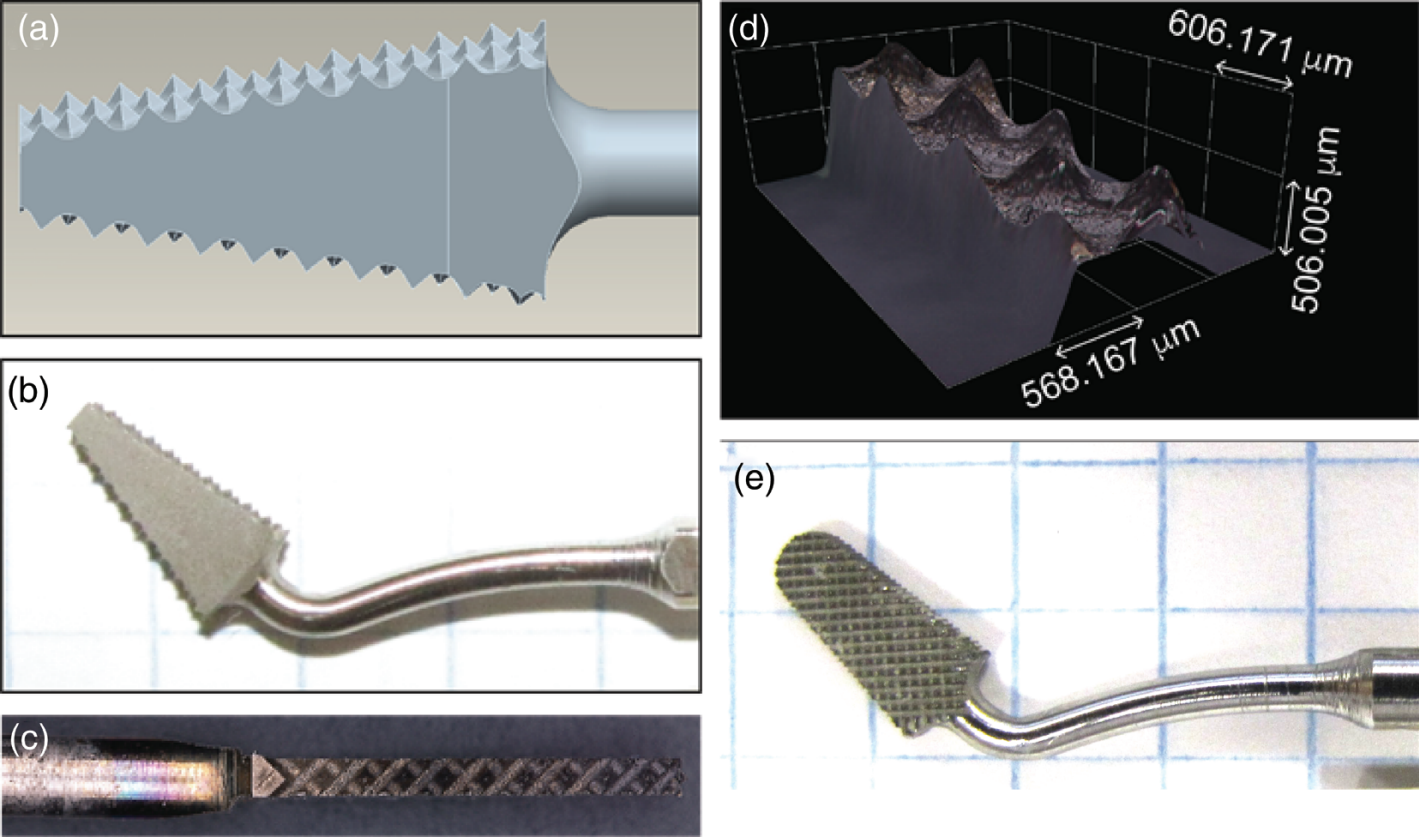
Design, fabrication, and characterization of implant‐shaped piezotome roughing and finishing tools. (a) Roughing tool CAD. (b) Roughing tool face and neck photograph. (c) Photograph of roughing tool cutting edge. (d) Confocal laser scanning micrograph of roughing tool teeth. (e) Finishing tool photograph

Standard state‐of‐the‐art implants are typically based on cylindrical screws that require round holes cut by cylindrical burs. Such a circular cross section is not suitable for the unique trapezoidal shape of the PiezoImplant. Thus we set out to design and fabricate a tapered, rectangular cutting tool to ensure tight apposition of bone and implant during future osseointegration processes. Our tool is based on a piezoelectric platform as piezotomes have been shown to have lower cutting forces that reduce secondary fracturing of bone while still producing accurate cuts (Zhang et al., 2017). Design specifications for the novel tool head included matching the tool geometry and neck angle to the implant and instrument while reducing tool oscillations that may disrupt cutting. The final shape was a product of optimizing via finite element analysis an elliptical movement with maximum amplitude of the tip at the piezotome's working frequency of 30 kHz (Figure 5). This finite element analysis refinement of tool shape was required to achieve a sawing motion. The optimization starting point was a straight tool that is exposed to an axial movement caused by the piezo stack inside the piezotome but did not cut. The initial simulations demonstrated that the starting straight design of the tool does not lead to any magnification of the tip displacement compared with the excitation of the piezo stack. This is because the first natural mode, which would lead to a magnification, has an Eigenfrequency of roughly 47 kHz (working frequency of the piezotome is 30 kHz). Improving ultimate cutting performance was included in the simulation by optimizing a vibrational mode that allowed the tool tip to vibrate in an elliptical mode when the tool is axially excited by the piezo stack. The final design simulation (Figure 5) highlights both the final tool geometry and the maximum deflection and deformation for the given excitation by the piezotome (3‐μm axial displacement with a frequency of 30 kHz.) The PiezoImplant roughing and finishing tools are shown Figure 6. Overall, the roughing tool has pyramidal cutting teeth on the lateral surfaces whose manufacture was verified by confocal laser scanning microscopy. The finishing tool matches the dimensions of the PiezoImplant and has cutting teeth across each face of the tool.

**Figure 7:**
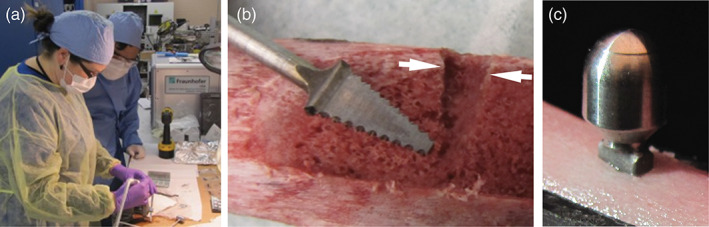
Testing of the piezoelectric cutting tools. (a) Periodontal surgical team using novel piezoelectric roughing tool to cut trapezoidal hole into pork rib bone model. (b) Cross section of hole with white arrows highlighting surface proximal edges of rough cut. (c) Implant with abutment seated in pork rib bone model

After manufacturing the piezoelectric tools, it was important to verify the geometry of the cut in bone material. Pork rib was selected for the initial cutting tests due to the ease of cross sectioning and availability. As shown in Figure 7, the custom manufactured tool heads for the PiezoImplant were able to accurately cut into the bone and generate the required geometry for ultimate implantation. This effort validated our finite element analysis for tool shape optimization (Chang, Tambe, Maeda, Wada, & Gonda, 2018).

On a qualitative level, the periodontal team experienced in piezoelectric‐based osteotomies indicated no substantial differences in terms of ease of use or cutting duration in this model. PiezoImplant sites are underprepared with the PiezoImplant tool, and the implant is tapped into the osteotomy in order to obtain primary stability. This is similar to existing cylindrical implant systems, where the osteotomy is underdrilled and the threaded implant is torqued in place. During our ex vivo testing of the PiezoImplant, we have obtained good primary stability and the implant could not be pulled out of the bone with hands.

In our future work, standardized bone material will be used to conduct a complete study on tool functionality including tool wear, cutting duration, and material temperature via infrared thermography in order to fully characterize our novel tool and its impact on bone (Mohlhenrich, Modabber, Steiner, Mitchell, & Holzle, 2015).

## 4 DISCUSSION

Atrophic jaws have long been a challenge to restoring tooth functionality. Dental implants can provide a robust and positive alternative to traditional denture‐based methods, but several difficulties remain. These include bone grafting to provide a wide enough base for cylindrical screw implants. One of the challenges is the amount of bone available for implant support in patients with long‐lost teeth. In the absence of a functional load, bone gradually resorbs in edentulous areas, resulting in vertical (shallow) or horizontal (narrow) ridge deficiency. In these cases, bone grafting has become the standard of care; however, the addition of the extra surgical procedure incurs additional surgical risk, treatment time, and cost. Short implants have been developed as alternatives to bone grafting in cases of vertical ridge defects. A systematic review comparing the placement of short implants versus bone grafting and placement of regular‐size implants showed similar implant success rated but higher surgical complication rates in the bone grafted group (Nisand, Picard, & Rocchietta, 2015). A Cochrane systematic review found borderline significant increase in implant failure rates and significantly more complications in vertically augmented sites versus short implants (Esposito et al., 2009). Thus, short implants can provide a viable alternative to bone grafting and have gained increased clinical acceptance in vertical bone defects. The PiezoImplant is designed to offer a similar alternative to bone grafting for horizontal bone defects, as it can be placed in narrow ridge without bone grafting.

Here, we have described the design and manufacture of rectangular implants with dimensions suitable for use in atrophic jaws without grafting. Although definitions in the literature vary, at 2.1‐mm wide, our PiezoImplant is categorized as extra‐narrow (<3.0 mm) (Al‐Johany, Al Amri, Alsaeed, & Alalola, 2017), a “mini‐implant,” or Category 1 narrow‐diameter implant (NDI) (Klein, Schiegnitz, & Al‐Nawas, 2014). Overall, NDI implants can be equally successful as standard‐diameter implants in terms of maintaining a marginal bone level and in overall success rates(de Souza et al., 2018). Although the most narrow of NDIs have lower survival rates than wider counterparts, meta‐analyses indicating success is still quite high (~92–95%)(Lemos et al., 2017; Schiegnitz & Al‐Nawas, 2018). A recent prospective cohort study of mini‐implants demonstrated that survival and success rates of the 20 patients were 100% after ~5 years.(Enkling et al., 2019) These studies suggest that narrow alveolar ridges can support implantation, keeping in mind that implant failure does occur depending on implant material, length, and location(Altuna et al., 2016; Jawad & Clarke, 2019; Van Doorne et al., 2019). Thus, further studies with larger populations and longer study periods with rigorous metrics of success are strongly encouraged.

Due to the rectangular geometry of our PiezoImplant, it is expected that when compared with extra‐narrow implants of standard cylindrical geometries (screws), the PiezoImplant will exhibit a higher level of stability. Rectangular cross sections have higher second moments of inertia (*I*) that reduces the possibility of bending and therefore failure (Morgan & James, 1995). This view is supported by the predicted reduced von Mises stresses (Figure 3) and the survival of the manufactured implant after 5 × 10^6^ fatigue cycles under a 340‐N load (Figure 4). The direct next steps for implant testing will be implantation into living bone to rigorously monitor osseointegration potential. These additional studies will be crucial to monitor safety and overall feasibility of our designs.

The rectangular cross section of the PiezoImplant also necessitated the de novo design of matching tooling. Design and manufacture of tool heads that were compatible with existing piezosurgery platforms indicated for oral surgery provided a straightforward route to production and testing. Piezoelectric tooling for implant site preparation is gaining in popularity. In general, the advantages of piezotools over conventional cylindrical burs involve greater control of the device and selective cutting of mineralized bone (Otake et al., 2018). A recent meta‐analysis also indicated that implant stability was greater for piezosurgeries when compared with cylindrical bur site preparation with marginal bone levels comparable for both groups (Atieh, Alsabeeha, Tawse‐Smith, & Duncan, 2018).With the bone and implant performing either the same or better during piezosurgeries, it continues to be a promising solution for osteotomies, where piezosurgery does not perform as well is in surgery time and tool robustness. For piezoelectric tooling, surgery times are typically longer, and tool tips break more frequently which work together to increase costs. Ultimately, implant site preparation for rectangular implants is difficult to imagine without piezotooling. We envision that the advantage of increased flexural rigidity for rectangular geometries and the ability to implant within a narrow atrophic jaw site make this approach promising. Indeed, increased surgery time and costs would likely be offset by eliminating bone grafting procedures.

Together, successful design and manufacture of an implant is a long process that integrates a number of fields of study and requires longitudinal studies in clinical testing. In this workflow, mechanical specifications (material choice, material surface roughness, material geometry, implant/abutment connection, etc.) compete with surgical requirements (ease of use, time of surgery, dimensions of bone, temperature at implant site, etc.). These considerations must be balanced with manufacturability and of course, the basic biology of osseointegration. Here, we have presented the beginning of this process for a novel implant design. Our direct next steps will be testing the tooling in standardized materials monitoring pressure, duration, and temperature so that we may compare with outstanding implants in the marketplace. We will additionally move to test implant stability in live bone to monitor osseointegration.

## AUTHOR CONTRIBUTIONS

R. G., S. D., and A. S. B. did the concept and design. H. W. and S. T. performed the data collection. All authors contributed to data analysis. S. T., C. M., and A. S. B. did the drafting of the article and the critical revision. All authors approve this article.

## Supporting information


**Table S1** Supporting Information
