# Supplementary material for: Design and ex vivo characterization of narrow implants with custom piezo‐activated osteotomy for patients with substantial bone loss
Source: Clin Exp Dent Res. 2019 Dec 31;6(3):336–44. doi: 10.1002/cre2.276 (PMC7301390; doi:10.1002/cre2.276)
Supplement: Supplementary file 1 — Table S1 Supporting Information [file CRE2-6-336-s001.docx]

**Supplementary Table 1.** 30° Compression Fatigue Test Results

| **Sample ID** | **Maximum Load (N)** | **Minimum Load (N)** | **Cycles to Failure** | **Failure Mode** |
| --- | --- | --- | --- | --- |
| 1446 | 340 | 34 | 5,000,000 | 1 |
| 2762 | 340 | 34 | 5,000,000 | 1 |
| 2964 | 340 | 34 | 5,000,000 | 1 |
| 2863 | 400 | 40 | 114,400 | 2 |
| 2360 | 400 | 40 | 590,500 | 2 |
| 1547 | 400 | 40 | 181,800 | 2 |
| 708 | 416 | 42 | 57,400 | 2 |
| 1036 | 416 | 42 | 865,000 | 2 |
| 1848 | 416 | 42 | 43,600 | 2 |
| 602 | 475 | 48 | 10,100 | 2 |
| 1337 | 475 | 48 | 9,800 | 2 |
| 2054 | 475 | 48 | 7200 | 2 |

1. No failure—test discontinued at 5,000,000 cycles
2. Implant crack below pot line (see **Figure 5B**)
